# Design of Experiments-Assisted Development of Clotrimazole-Loaded Ionic Polymeric Micelles Based on Hyaluronic Acid

**DOI:** 10.3390/nano10040635

**Published:** 2020-03-29

**Authors:** Laura Catenacci, Giorgio Marrubini, Milena Sorrenti, Silvia Rossi, Giuseppina Sandri, Franca Ferrari, Valentina Fagnani, Caterina Valentino, Maria Cristina Bonferoni

**Affiliations:** Department of Drug Sciences, University of Pavia, Viale Taramelli 12, 27100 Pavia, Italy

**Keywords:** hyaluronic acid, ionic polymeric micelles, design of experiments, hexadecyl amine, antifungal drugs, clotrimazole

## Abstract

Polymeric micelles based on amphiphilic polysaccharides have some advantages as a carrier of poorly soluble lipophilic drugs thanks to their characteristic "core–shell" structure. Previously, ionic polymeric micelles based on chitosan and fatty acids have been developed. The aim of the present study was the preparation and characterization of hyaluronic acid (HA) derivatives by direct ionic interaction between the HA carboxylic groups and the amine groups of dodecyl amine (DDA) and hexadecyl amine (HDA). The HA–HDA polymeric micelles were loaded with a poorly soluble hydrophobic antifungal drug, clotrimazole (CLO). A 2^3^ full factorial experimental design was used to evaluate the effect of the following factors: HA/HDA ratio from 1:0.25 to 1:0.75, cholesterol (CHOL%) as percentage of HA from 10% to 30%, and preparation temperature from 20 to 40 °C. As dependent variables (responses), nanoparticle dimensions and clotrimazole concentration in the final colloidal dispersion were considered. To optimize the drug final concentration, the design was therefore expanded into a rotatable central composite design (CCD). The effects of the formulation variables and the composition of the optimized formulation were confirmed by a mixture design. Physicochemical characterization of the optimized formulation was performed, confirming the ionic interaction between the polysaccharide and the HDA.

## 1. Introduction

The use of amphiphilic derivatives of bioactive polymers in the preparation of polymeric micelles presents peculiar advantages for the delivery of poorly soluble lipophilic active principles, such as those belonging to class II and IV of the Biopharmaceutical Classification System whose bioavailability is impaired by low solubility [1]. In aqueous environment, in fact, lipophilic portions of the amphiphilic polymer self-assemble into domains that can accommodate hydrophobic payloads. Polymer hydrophilic chains arrange to form the polymeric micelle outside shell, toward the aqueous environment and the interface with the biological substrates. Therefore, polymeric micelles can maintain the peculiar properties of interaction with the biological substrates that characterize the original bioactive polymer. 

In the case of hyaluronic acid (HA), among its documented biological activities, antiangiogenic and anti-inflammatory effects can be remembered in the case of high molecular weights. Pro-inflammatory, angiogenic, and immune-stimulatory activity is claimed in the case of low molecular weights (<100 kDa) [2,3]. The literature largely described the polymer ability to interact with the cluster of differentiation protein CD44, which is a receptor that is physiologically involved in regulating cell adhesion and migration and is overexpressed in different kind of tumors. This behavior suggested the use of HA nanocarriers for tumor targeting [4,5]. Recently, some authors suggested the interaction of HA with CD44 receptors as a mechanism of penetration of nanoparticles coated with HA, which was proposed to deliver amphotericin B to vaginal mucosa for vulvovaginal candidiasis [6]. The HA ability to interact with skin stratum corneum was studied to elucidate the mechanisms of the observed penetration enhancement properties. Low molecular weight HA (5 kDa) was able to improve penetration in intact skin even of a macromolecule such as bovine serum albumin, thanks to stratum corneum hydration, interaction with keratin, and cotransport of the protein together with HA. In the case of tape-stripped skin, higher molecular weight HA (100 kDa) showed interaction with stratum corneum lipids and the highest improvement in HA penetration [7]. 

The literature is rich in examples of the hydrophobic modification of polysaccharides such as chitosan and HA by covalent derivatization to obtain amphiphilic derivatives that are able to form polymeric micelles by spontaneously self-assembling. This occurs by linking acidic moieties on chitosan amino groups to obtain amidic functions [8]. In the case of HA, the polymer carboxylic functions have been exploited to link hydrophobic moieties such as aminoethyl 5β-cholanoamide [9]. HA was conjugated with octadecyl amine by the interaction of amino groups of octadecyl amine with carboxyl groups of HA through carbodiimide and N-hydroxy succinimide activation and was proposed as a carrier for the oral delivery of paclitaxel [10]. Similarly, HA acyl derivatives with chains of different lengths were recently synthesized and deeply characterized with respect to their physicochemical properties [11]. 

Among acyl derivatives, oleoyl, capryl and hexyl HA derivatives have been studied and evaluated for their biocompatibility and cell interaction. In particular, the efficiency and the mechanisms of penetration enhancement of these derivatives in the skin have been elucidated [12,13,14].

Previously, in our research group, polymeric micelles based on chitosan palmitate conjugates have been assessed for ocular delivery of cyclosporine [15]. Ionic chitosan derivatives based on chitosan and fatty acids salts have been developed for the delivery of poorly soluble drugs in wound healing [16,17] and oral delivery [18]. The aim of the present study was the assessment of an analogous approach for the preparation and characterization of HA ionic polymeric micelles based on amphiphilic polymer derivatives, which are obtained by ionic interaction between the carboxylic groups of the polysaccharide and the amine groups of long-chain amines such as hexadecyl amine (HDA). The choice of HDA was encouraged by the previous experience with chitosan palmitate nanoparticles [15]. The obtained polymeric micelles were loaded with the poorly soluble hydrophobic antifungal drug clotrimazole (CLO). 

The effect on micelle preparation of some formulation variables such as the ratio between polysaccharide chain and hydrophobic counterion, the cholesterol (CHOL) presence, and preparation temperature have been studied employing a Design of Experiments (DoE) approach. This involves screening designs aimed to clarify the effects of some parameters by checking them at two levels, and response surface designs, based on the evaluation of the parameters at three levels, which are useful to identify the values that have to be selected to obtain the desired response. This demonstrates evidence of the relevance of interactions between the factors and highlights the significant effects on a statistically sound basis [19,20]. Some papers are present in the literature using DoE in the development of nanomedicines [21] and more specifically of polymeric micelles. Some of them refer to systems based on poloxamers [22,23], and in a few cases, HA-based polymeric micelles have been developed [24]. In the present work, in a first phase, a screening full factorial design 2^3^ was used, and as response (dependent) variables, nanoparticles’ dimensions and CLO concentration in final colloidal dispersion were considered. Therefore, the CLO colloidal concentration was optimized by a response surface central composite design (CCD). The results of this part of the study were confirmed by using a mixture design, in which the composition of mixtures of HA, HDA, and CHOL was investigated. This allowed the identification of the optimized nanoparticle formulation that was therefore characterized in terms of physicochemical properties such as particle dimensions and zeta potential, and analyzed by FTIR spectroscopy and thermal analysis to identify a possible interaction between the components in the polymeric micelles.

## 2. Materials and Methods 

### 2.1. Materials

Low molecular weight Na hyaluronate was used (Bioiberica, Barcelona, Spain), which was measured by capillary viscosimetry as about 50 kDa. Dodecyl amine (DDA), hexadecyl amine (HDA), and cholesterol (CHOL) were from Sigma-Aldrich (Milan, Italy). Pyrene was from Fluka (Milan, Italy). Clotrimazole (CLO) was obtained from Sifavitor (Casaletto Lodigiano, Lodi, Italy). Ketoconazole was used as an internal standard and obtained from Erregierre (San Paolo D’Argon, Bergamo, Italy). All the other reagents were from Carlo Erba (Milan, Italy).

### 2.2. Polymeric Micelles Preparation

To prepare polymeric micelles, solutions of DDA or HDA in acetone were added dropwise to a 0.05% w/v solution of Na hyaluronate in filtered distilled water. The amount of DDA or HDA has been calculated based on the stoichiometric ratio between the carboxylic groups of HA and the amino groups of DDA or HDA. Acetone was removed under nitrogen flux at room temperature. For the loaded systems, either pyrene or CLO was added dissolved in acetone together with the amine and, when present, with CHOL. The CLO final theoretical concentration was 83 µg/mL. In the case of pyrene, the theoretical final concentration was 1 × 10^−7^ M. The colloidal dispersion of polymeric micelles was separated from the not encapsulated poorly soluble drug (or pyrene probe) through a 10-min 6000 rpm centrifugation step. 

### 2.3. Polymeric Micelles Characterization

#### 2.3.1. Dimensional Analysis and Zeta Potential 

The dimensional analysis of the micelles was carried out using Photon Correlation Spectroscopy (PCS) by an N5 Submicron Particle Size Analyzer (Beckman Coulter, Milan, Italy). Dilutions of the samples were performed in distilled filtered water. The optimized formulation was assessed in CH_3_COOH/CH_3_COONa buffer 0.1 M at pH 4.0, 5.0, and 6.0. Three replicates were performed for each sample. Zeta potential was evaluated by a Zetasizer® nano series (Malvern Instruments Ltd., Worcestershire, UK) in aqueous suspension.

#### 2.3.2. Pyrene Spectra 

Pyrene solubilized in water and encapsulated in polymeric micelles was analyzed by a spectrofluorimeter (Perkin Elmer, LS 50B, Milan, Italy) at *λ*_exc_ = 336 nm. The emission spectrum was considered between 350 and 500 nm and the ratio of the intensity at *λ*_em_ = 383 nm and *λ*_em_ = 372 nm was calculated according to what described by Kalyanasundaram and Thomas [25].

#### 2.3.3. HPLC-UV Determination of Clotrimazole in the Formulations 

The concentration of CLO encapsulated in the micellar systems of interest was determined by an HPLC-UV method preceded by the extraction of the active ingredient from the sample. To 1 mL of sample, 25 μL of 0.6% w/v ketoconazole in acetone was added as the internal standard to obtain a final concentration of 150 μg/mL. The acetone added was evaporated completely under nitrogen flow at room temperature. To facilitate the extraction of the encapsulated CLO, the micelles were broken by adding 1 mL of 0.1 M aqueous NaOH and stirring in Vortex about 1 min. Liquid–liquid extraction was performed by using 4 mL of ethyl acetate by Vortex stirring for 5 min and separation by centrifugation at 3000 rpm for 30 min. Then, 2 mL of the organic phase was taken and dried under nitrogen flow. The sample was finally resuspended with 250 μL of mobile phase, and 50 µL of this solution were injected into the HPLC system. The HPLC method involved the use of a C18 column (4.6 × 150 mm–5 μm particle size—80Å porosity) and a mobile phase consisting of MeOH/K_2_HPO_4_ 0.025 M in a 75:25 (v/v) ratio. The analytes detection was at 261 nm, which was the maximum of absorbance of CLO in the mobile phase used. The concentration of CLO in the micelles was evaluated using a three-point calibration line obtained by adding 250–100–6.25 μg/mL of CLO to samples containing 150 μg/mL of the internal standard. The calibration line was obtained by least-squares ordinary regression of the ratio of the areas of the peaks of CLO and ketoconazole versus the ratio of the concentrations of CLO to ketoconazole. 

### 2.4. Design of Experiments

#### 2.4.1. Full Factorial Screening Design

Two responses were considered: the concentration of encapsulated CLO (Y1) and the mean particle size (Y2). Three factors were studied. X1 was the stoichiometric molar ratio between HA and HDA ranging from 1:0.25 (level coded as −1) to 1:0.75 (level coded as +1). X2 was the amount of CHOL loaded, selected as wt % of the amount of HA, ranging from 10% (−1) to 30% (+1). X3 was the micelles preparation temperature, ranging from 20 (−1) to 40 °C (+1). The design involved a total of 2^3^ = 8 experiments. Additional independent experiments were planned in the central point of the experimental domain (Table FFD, level 0 for each factor). 

#### 2.4.2. Central Composite Design and Mixture Design

Following the screening phase of the study, a CCD was built adding the star points to the previous full factorial design, as reported in Table S1 (Supplementary Materials). The CCD study was aimed at the assessment of the conditions for the preparation of the micelles to optimize the concentration of CLO as a colloidal solution. A mixture design of nine additional experiments was also used to study the response represented by the concentration of CLO. For this latter design of the experiments, given the results obtained in the previous studies, the operating temperature was fixed at 30° C. The three components of the mixture design are reported in Table 1, together with their constraints (composition limits).

The analysis of the data was performed by Statgraphics Centurion XVI software (2017 Statgraphics Technologies, Inc., The Plains, VA, USA) for the full factorial and the CCD. The design and evaluation of the mixture design were performed using Design-Expert® version 7.0.0 software (Stat-Ease Inc., Minneapolis, Minneapolis, MN, USA).

### 2.5. Physicochemical Characterization sxw2

#### 2.5.1. Fourier Transform Infrared Spectroscopy (FTIR) Analysis

IR spectra were recorded using a Fourier transform infrared spectrophotometer (Perkin Elmer SpectrumOne, Monza, Italy) with a single reflection ATR accessory (PIKE MIRacle^TM^). The samples were placed on an ATR crystal of ZnSe. The spectra were collected with a resolution of 4 cm^−1^ within the spectral range of 650–4000 cm^−1^.

#### 2.5.2. Differential Scanning Calorimetry (DSC) and Thermogravimetric (TGA) Analysis

DSC curves were recorded using a Mettler STAR system (Mettler Toledo, Milan, Italy) equipped with a DSC821^e^ Module and an Intracooler device for sub-ambient temperature analysis (Julabo FT 900) on 2–3 mg (Mettler M3 Microbalance) samples placed in sealed aluminum pans with a pierced lid, in the heating range of 30–300 °C [heating rate β = 10 K·min^−1^, nitrogen air atmosphere (flux 50 mL·min^−1^)]. High purity indium metal was used as a standard reference for the instrument calibration. Measurements were carried out at least in triplicate. Mass losses were recorded with a Mettler STAR^e^ system (Mettler Toledo, Milan, Italy) TGA with simultaneous DSC (TGA/DSC1) on 3–4 mg samples in alumina crucibles with a lid [heating rate β = 10 K·min^−1^, nitrogen air atmosphere (flux 50 mL·min^−1^), 30–300 °C temperature range]. The instrument was previously calibrated with indium as a standard reference and measurements were carried out at least in triplicate.

## 3. Results and Discussion

### 3.1. Comparison of the HA/DDA and HA/HDA Systems

**Figure 1:**
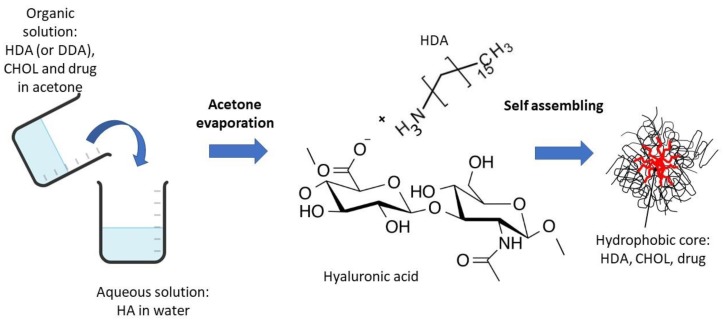
Scheme of the preparation of ionic polymeric micelles.

Table 2 shows the values of mean dimensions (particle size, PS) and polydispersion index (PI) of ionic polymeric micelles obtained by the interaction between HA and two long-chain amines, DDA and HDA according to the scheme illustrated in Figure 1. The long-chain amines were used in a stoichiometric ratio of 1:1 to the monomeric units of the HA. The table shows the data obtained by PCS at a 90° scattering angle for the unloaded and pyrene-loaded micelles. In the case of the unloaded micelles, no significant differences were observed between the systems obtained with the two different amines, which both showed dimensions slightly above 400 nm. In the case of loaded systems, smaller micelles of about 200 nm, with a lower PI were obtained using HDA as hydrophobic moiety. This is in line with the results obtained with analogous systems [15]. It is supposed that the presence of the payload has a stabilizing effect on the hydrophobically driven self-assembly of the amphiphilic polymers.

Pyrene is a probe characterized by high hydrophobicity. It is highly sensitive to the polarity of the surrounding environment, and when encapsulated in a hydrophobic environment, as in the core of micellar systems, it undergoes a spectrum modification. The spectrofluorimetric analysis records the intensity of characteristic vibrational bands of the pyrene, the ratios of which vary depending on whether the fluorescent probe is in an aqueous environment or is affected by a hydrophobic microenvironment [25]. To prove the formation of hydrophobic domains in polymeric micelles, the recording of the ratio between the intensities of the peaks of the pyrene spectrum at about 383 nm and 372 nm (R_III/I_) was suggested in the literature. It was reported that this ratio increased when the pyrene molecule was surrounded by a more hydrophobic environment compared to the same ratio in water [16,25]. In the present study, the loading of micellar systems with pyrene aims to demonstrate that the interaction between carboxylic groups of HA with the amino groups of DDA and HDA leads to the formation of carriers with domains in which hydrophobic molecules can be accommodated. 

Table 3 shows the intensity values of the peaks and their relative ratios for the different micellar systems. An increase in the I_372_/I_383_ ratio can be observed for both systems but results more marked in the case of the interaction product with HDA, which is conceivably due to the greater hydrophobicity induced by the chain with a higher number of carbon atoms.

This result, together with the comparison of the dimensional characteristics, suggested the choice of the HA/HDA system to continue the study.

### 3.2. Design of Experiments Analysis 

#### 3.2.1. Full Factorial Screening Design 

**Figure 2:**
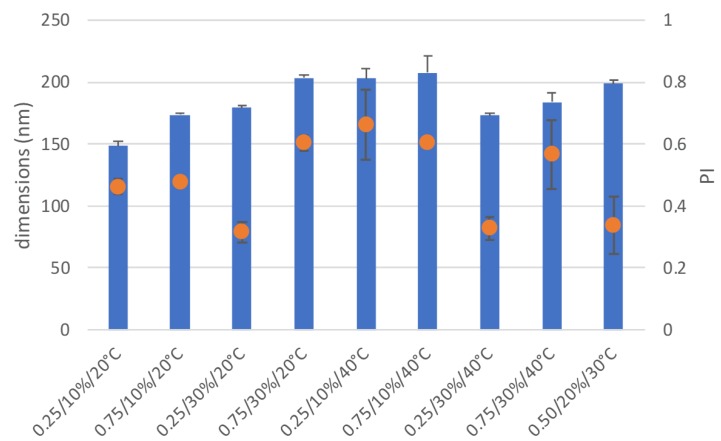
Dimensional characterization of the samples of the full factorial design. Dimensions (bars) and polydispersion index (PI, round symbols). (mean ± sd, n = 3).

The dimensional characterization of the micelles corresponding to the different runs of the full factorial 2^3^ screening design is illustrated in Figure 2. The mean diameters are given together with the PI index values. The micelle dimensions were in all cases within a narrow range of small values, between 148 and 207 nm. This result was in line with the ANOVA analysis reported in Table 4, showing that none of the studied factors, within the considered levels, had a significant effect on the particle dimension response. The poorly relevant effect of the degree of substitution on the system dimensions seems not in line with what was observed in the case of micelles based on chitosan palmitate conjugates [15,26]. An explanation can be found in the quite low molecular weight of the polymer used in the present work and in the structure of HA less rigid than that of chitosan chains. The overall effect of temperature was not statistically significant, although at least for low CHOL levels, temperature determined an increase of particle dimensions. This could be explained with a less ordered structure of hexadecyl chains in the micelle core at the higher temperature. The relationship between CHOL% and temperature appeared statistically significant, suggesting that the increase in CHOL% induced a moderate increase in particle size at the lowest temperature. On the other way, it was possible to avoid the increase in size due to the effect of the higher temperature by keeping the CHOL% at its higher level of concentration. This observation is of a limited impact considering the low variations in dimensions, but it can suggest a stabilizing effect of CHOL on the micelles, conceivably because it favors the self-assembling of the hexadecyl chains in the hydrophobic inner core.

**Figure 3:**
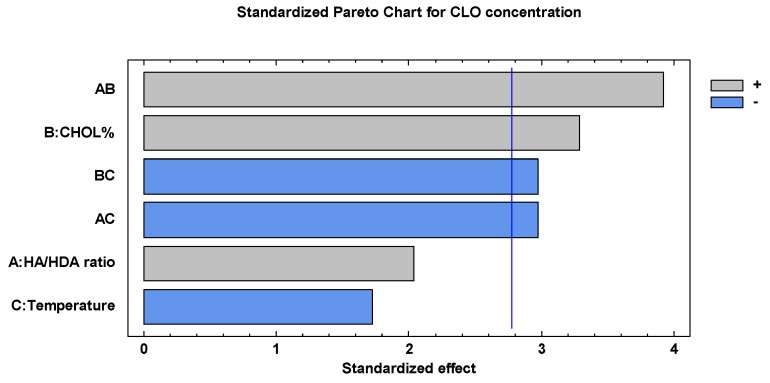
Standardized Pareto chart of the effects of the studied factors and their interactions on the CLO concentration in micelle dispersion.

**Figure 4:**
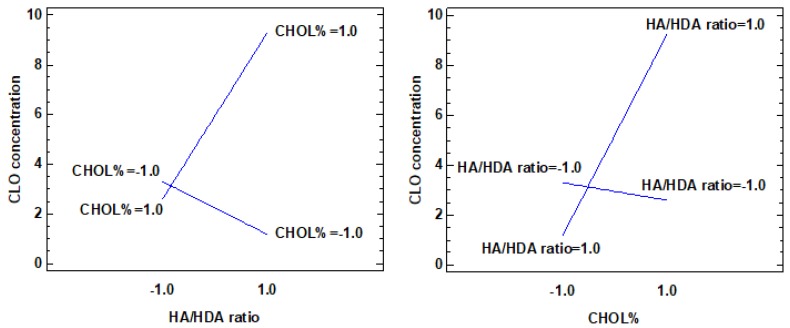
Interaction plot of CHOL% and HA/HDA ratio on CLO concentration.

Figure 3 and Table 5 show the results of the analysis of the full factorial design considering the concentration of CLO in the micellar system as the response variable. Among the primary factors, only the concentration of CHOL seems to significantly influence the ability of the micellar systems to load the drug and to increase its concentration. The CHOL% positive function for the stabilization of the system’s hydrophobic core, as previously observed for the particle dimensions response, seems therefore here confirmed. The HA/HDA ratio has a positive although not statistically significant effect. However, it is conceivable that the ability of the micelles to load the hydrophobic drug is related to the greater relative quantity of hydrophobic chains in the polymeric derivative. The importance of the HA/HDA ratio is highlighted by the fact that the loading of the CLO is more efficient when the increase in CHOL% occurs in systems prepared with the highest levels of HDA substitution, as illustrated by the interaction plot reported in Figure 4.

#### 3.2.2. Central Composite Design

To better investigate the effect of the factors that are being studied, and especially of the HA/HDA parameter on CLO colloidal solubilization, the full factorial design was expanded to a CCD response surface design. In this case, a quadratic model is suitable to interpret the experimental data, predicting the CLO concentration for each factor level [20].

**Figure 5:**
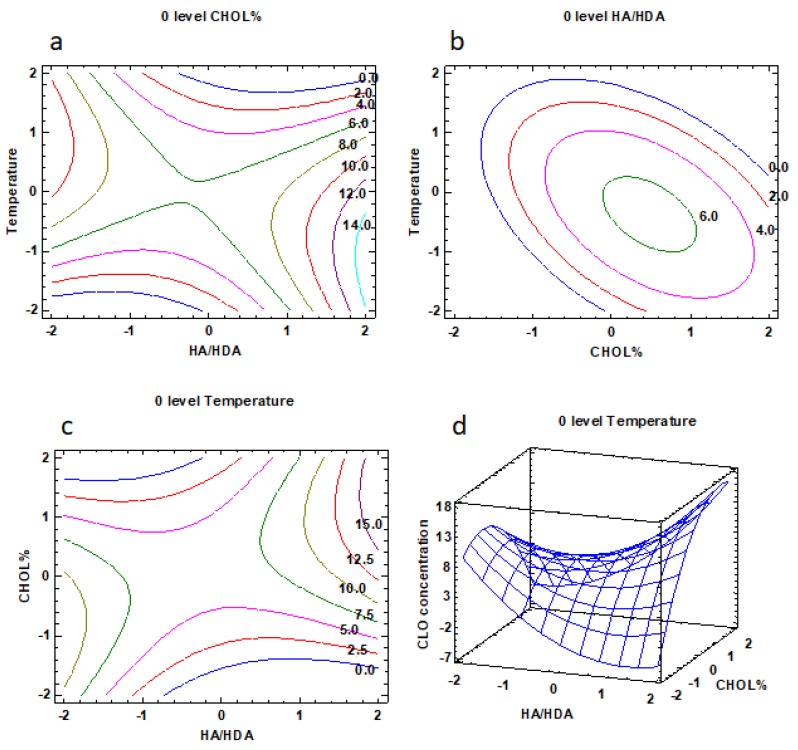
Central composite design results. CLO concentration (µg/mL) depending on temperature and HA/HDA factors (**a**), on temperature and CHOL% factors (**b**), and on CHOL% and HA/HDA factors (**c**,**d**).

In Figure 5, the results of the CCD design are illustrated, while the experimental plan and the obtained data of CLO concentrations are reported in Table S1 in the Supplementary Materials file. 

The fitted quadratic model equation with the calculated coefficients for the factors, their interactions, and the quadratic terms is given here below [20]:

CLO concentration = 6.15 + 0.65 × HA/HDA + 1.1544 × CHOL% – 0.40 × Temperature + 1.36 × HA/HDA^2 + 2.20 × HA/HDA × CHOL% − 1.67 × HA/HDA × Temperature – 1.83 × CHOL%^2 – 1.67 × CHOL% × Temperature – 1.65 × Temperature^2

R^2^ = 90.102%, adjusted R^2^ = 78.966%

In particular, in Figure 5 a–c, the iso-response curves are given with one of the factors maintained at zero level. Figure 5d illustrates as an example a three-dimensional response surface plot. In Figure 5a, the CLO concentration as a function of temperature and HA/HDA ratio (CHOL% level set to 0) is illustrated, showing that the highest CLO concentration solubilized (about 14 µg/mL) can be obtained at the highest levels of HDA substitution, which is close to the 1:1 stoichiometric ratio. Therefore, the positive effect of hydrophobic substitution along HA chains, as previously observed in the screening phase, is here confirmed. In line with this result, Figure 5b shows that at the 0 level of the HA/HDA ratio, that is the 1:0.5 stoichiometric ratio, a maximum CLO concentration of only 6.0% was attained. From Figure 5c,d, it is possible to see that the combination of high levels of CHOL% with high levels of HA/HDA ratio corresponded to a CLO concentration up to 15 µg/mL, confirming the positive interaction observed between these two factors in the full factorial design. 

#### 3.2.3. Mixture design

To confirm these results and find the best combination between the three components of the micellar carrier, a mixture design was used, with the constraints reported in the Methods section (Table 1). 

According to the previous CCD analysis, the maximum amount of CLO incorporated was predicted to be obtainable at the temperature of 13 °C, but this condition is not easily set for routine purposes, and therefore, the operating temperature of 20 °C was selected as the best compromise between reasonable preparation conditions and the optimal capacity of CLO encapsulation. 

**Figure 6:**
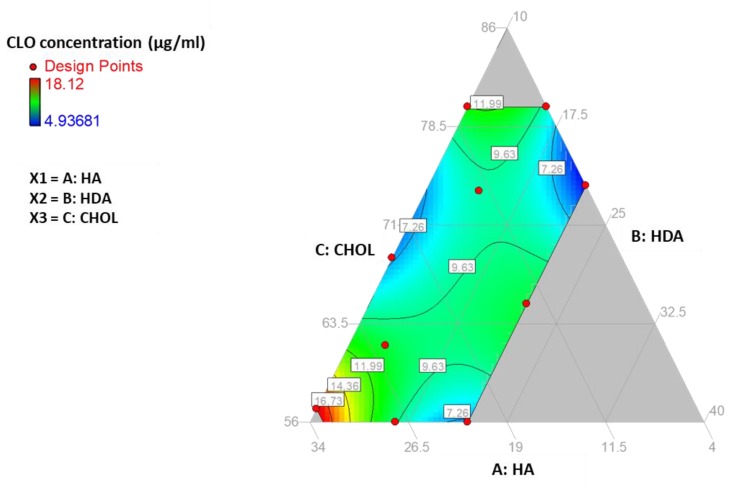
Mixture design results. CLO concentration (µg/mL) depending on CHOL, HA, and HDA fractions in the micelle composition.

The mixture design data are reported in Table S2 of the Supplementary Materials file. In Figure 6, the iso-response curves are reported. Combinations indicated by the red area correspond to the highest predicted CLO concentrations.

The model selected was the special cubic Scheffé model [27]. The mixture experimental plan was constructed in the domain defined by the minimum and maximum levels for each component and considering the mixture constraint, which fixes the sum of the amounts of the three components to the value of 1. Given the constraints of the three factors and the fact that the resulting experimental domain was an irregular polygon (Figure 6), it was not possible to use symmetric mixture designs, and it was necessary to use a D-optimal mixture design [27,28].

The software Design Expert® (Stat-Ease Inc., Minneapolis, MN, USA) proposed as the optimal choice the experiments in the domain points indicated in the figure with red points, which are respectively 5 of the 6 vertices of the polygon identified within the Scheffé simplex, 3 points on the longest sides, and 1 point inside the domain (Figure 6).

The three components considered were HA (component X1 ranging in fraction from 0.560 to 0.800), HDA (component X2, ranging in fraction from 0.040 to 0.330), and CHOL (component X3, ranging in fraction from 0.100 to 0.220). Thus, the percent amounts of the three components were tightly constrained as reported in Table 1.

The Scheffé model equation used is given here below:

CLO concentration = 51.64·HA + 286.95·HDA + 350.62·CHOL − 463.41·HA × HDA − 635.28·HA × CHOL − 551.35·HDA × CHOL

R^2^: 0.944, Adjusted R^2^: 0.851, p-value 0.04

The preference for the quadratic model was driven by the observation that the ANOVA of the regression was significant for this model, while it was not statistically significant for the special cubic model (p-value 0.06), and that therefore there was a non-negligible probability that the special cubic model could be severely affected by random noise.

Five additional independent experiments, each one carried out in two replicates, were performed on different days during the following weeks for the model validation. These results are listed in Table 6. All the observed results are in good agreement with the predicted ones. Therefore, the validation results show the usefulness of the mixture model computed, which is suitably predictive of the CLO concentration depending on micelle composition.

By using the model prediction and response surface plot, it could be concluded that the region of the experimental domain of maximum CLO colloidal solubilization was located in the surroundings of the mixture with a composition of 0.57 HA, 0.33 HDA, and 0.1 CHOL (Figure 6). The CLO concentration for this micelle composition is reported in Table 6, as one of the validation points (evidenced in bold). The CLO concentration observed, equal to 18.37 µg/mL, corresponds to a 36-fold increase compared to the CLO solubility in neat water, which is about 0.5 µg/mL according to the literature [29,30]. The results of the mixture design quite well agree with what was observed in the full factorial and the CCD. The best CLO solubilization can be obtained with a high HDA substitution, which for the optimal point corresponds to a molar ratio HA:HDA of 1:0.93, while the optimal amount of CHOL was confirmed as slightly higher than 20% of the HA amount. In general, high hydrophobic substitution and CHOL presence support the micelle stabilization and loading capacity.

As defined in the literature [31], the effect of changing the mixture composition was studied considering the different concentrations of CLO encapsulated passing from an arbitrary reference point composition to compositions in which every component was changed by 0.01, while keeping the other two remaining components at a constant relative ratio equal to that existing in the reference point composition. By doing so, it could be evidenced that the most important effect on the response was obtained by changing the component HDA (increase in the response of about 11% for an increase in the component fraction of 0.01); a positive effect appears evident for the increase of CHOL, and only an HA increase involves a decrease of CLO concentration. 

### 3.3. Physicochemical Characterization of the Optimized Micelle Formulation

The optimized micelle formulation was further characterized, both for the unloaded and the loaded polymeric micelles by measuring the zeta potential that resulted in both cases negative, which was equal to −31.43 (±0.50) mV for the unloaded micelles and −34.83 (±0.32) mV for the loaded ones, as expected considering the anionic nature of the HA. 

**Figure 7:**
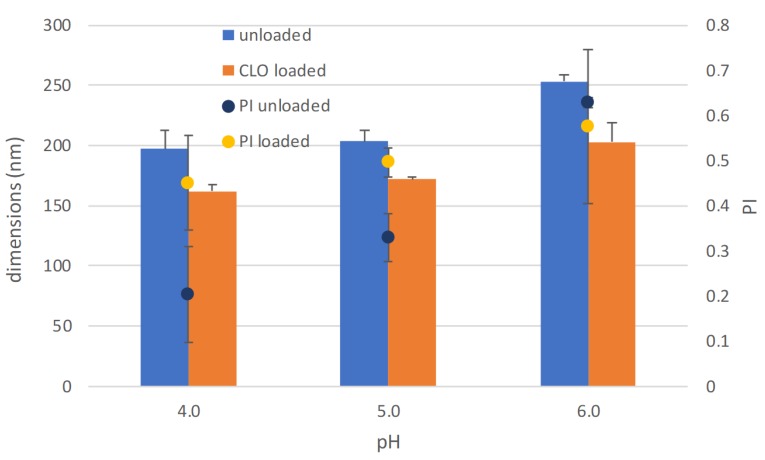
Dimensional characterization (bars) and polydispersion index PI (round symbols) of the optimized micelles, both unloaded and CLO-loaded, in different pH buffers.

In Figure 7, the dimensional characterization of the optimized polymeric micelles is illustrated both for the unloaded and for the CLO-loaded systems. The quite small dimensions previously seen for this kind of system are here confirmed. Moreover, the results of the dimensions were quite independent of the pH of the buffer in which the samples were diluted. Although this aspect would deserve more and deeper investigation, this preliminary result would suggest that the structure of the system, although based on ionic interactions, is quite stable in the range of the pH values evaluated, which encompasses the physiological pH of the skin and that of the vaginal environment [32,33]. It should be considered that this range is between the pKa values of 3 and 4 for HA and of about 10 for HDA. Both of the species interested in the ionic interaction are therefore mainly in the ionized form.

**Figure 8:**
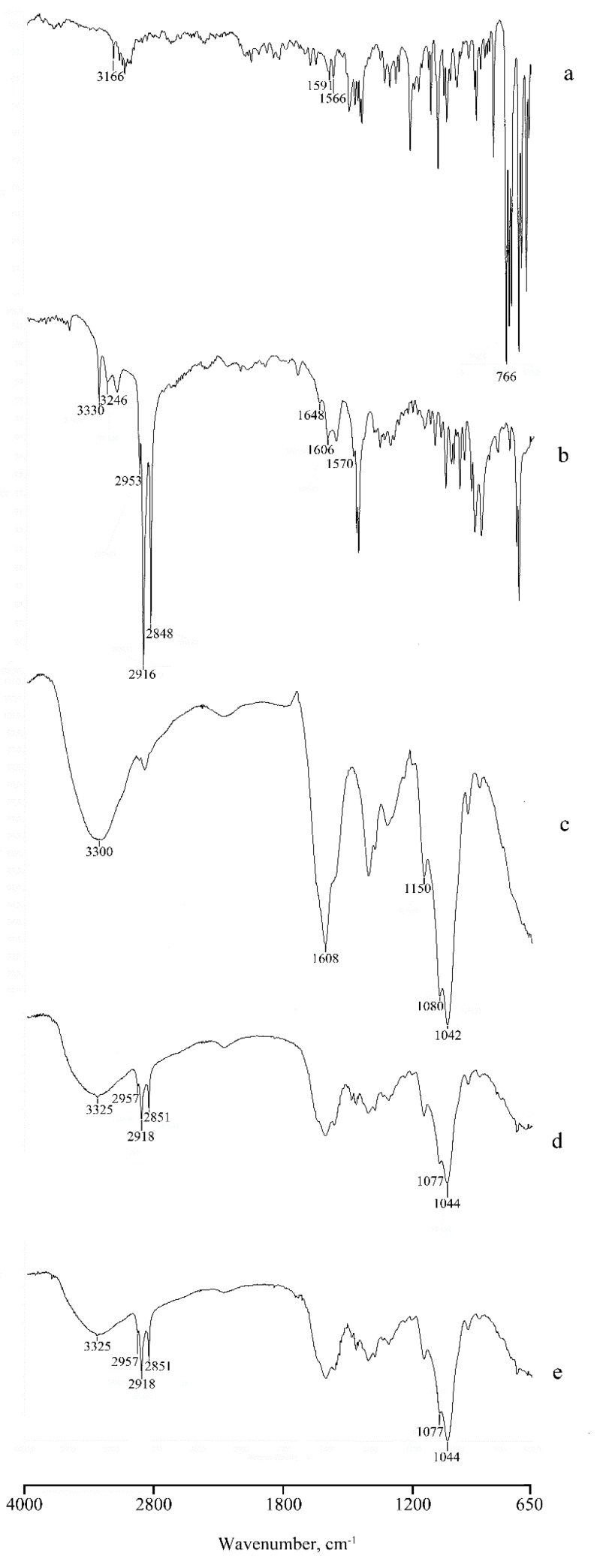
Fourier Transform Infrared Spectroscopy (FTIR) spectra of the pure drug (**a**), HDA (**b**), HA (**c**) and micelles, unloaded (**d**) and CLO-loaded (**e**).

The FTIR spectra of pure CLO, HDA, HA, and micelles, both unloaded and CLO-loaded, are shown in Figure 8. 

The pure CLO characteristic absorption bands are the aromatic C–H stretching at 3166 cm^−1^, the aromatic C=C stretching at 1591 cm^−1^, the C=N stretching at 1566 cm^−1^, and the aromatic C–H bending at 766 cm^−1^, respectively (spectrum a).

The FTIR spectrum of HDA (spectrum b) presents two bands at 3330 cm^−1^ and 3246 cm^−1^ that are characteristic of the primary amine stretching, three bands at 2953, 2916, and 2848 cm^−1^ of the CH_2_ stretching. In the region between 1650 and 1570 cm^−1^, three bands due to NH bending are present, too.

In the HA spectrum (spectrum c), the absorption band at around 3300 cm^−1^ corresponds to the 

–OH group, and the band at 1608 cm^−1^ is due to symmetric absorption from the –COO group. The bands at 1150, 1080, and 1042 cm^−1^ are typical of the –C–O–C group stretching.

The interaction between the two components in the unloaded micelle system (spectrum d) is revealed by the shift of some of these bands, in particular for the HDA the bands at 3325, 2957, 2918 and 2851 cm^−1^, and for HA the bands at 1077 and 1044 cm^−1^. The spectrum of micelles loaded with the drug (spectrum e) is superimposable to that of unloaded micelles, suggesting that the presence of CLO does not modify the stability of the systems. The typical bands of the drug are in this case hardly visible due to the low drug loading (about 2% w/w) and the overlap with the polymer bands.

**Figure 9:**
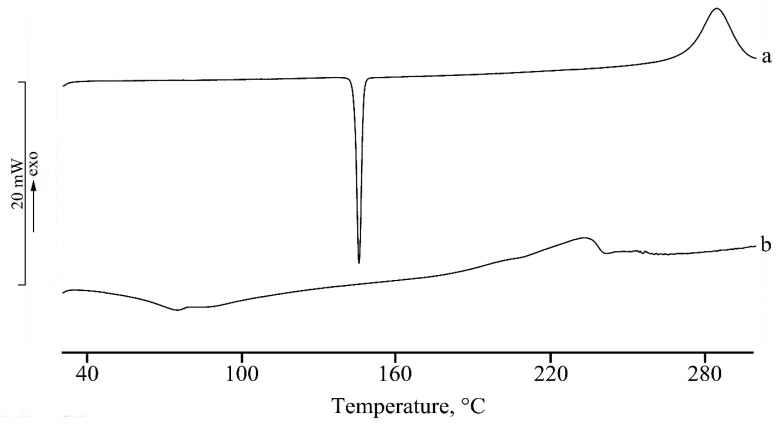
Differential Scanning Calorimetry (DSC) curves of the CLO (curve a) and of the CLO-loaded micelles (curve b).

The DSC behavior of the pure drug and the loaded micelles is reported in Figure 9. The crystalline anhydrous nature of CLO is revealed by DSC profile where an endothermic effect due to drug melting is registered at 144.7 ± 0.3 °C (ΔH_m_ = 98 ± 1 J·g^−1^), followed by sample decomposition from about 250 °C (Figure 9, curve a). The anhydrous state is also confirmed by the absence of mass losses in the temperature range from 30 °C to melting temperature in the TGA curve (data not reported).

The DSC curve of the CLO-loaded micelle systems (curve b) shows the thermal profile typical of an amorphous sample, suggesting the amorphization of the drug in the core of the micelles, although, considering the quite low drug loading, it may be difficult to identify the presence of the drug melting in the system. The profile that resulted was superimposable to that of unloaded micelles. The broad endothermic effect recorded in the temperature range between 60 and 100 °C is due to the absorbed water loss during heating, as confirmed also by TGA measurements (data not reported).

## 4. Conclusions

The data obtained confirmed that the ionic interaction between HA and a long-chain amine such as DDA or HDA resulted in an amphiphilic polymer that is able to self-arrange in the aqueous environment. Carriers in the nanometric range and with hydrophobic domains, which are characteristics of polymeric micelles, could be obtained. The application of the experimental design approach allowed us to get a deep knowledge of the system under study. The full factorial design put in evidence that the dimensions of the polymeric micelles were quite independent of the three factors studied, namely HA/HDA ratio, CHOL%, and temperature, and they remained in a narrow range between about 150 and 200 nm. The colloidal solubility of CLO associated with the polymeric micelles was influenced especially by the presence of CHOL and by the interactions between the studied factors. The expansion of the model to a central composite design confirmed the relevance of HDA substitution level and CHOL presence for the colloidal solubilization of the drug. The CDD results suggested setting the preparative parameter temperature at the level that represented the best compromise between reasonable practical conditions and the highest CLO concentration. The mixture design confirmed the findings of the previous studies and led to the finest choice of formulation parameter levels. The CLO concentration obtained with the optimized formulation was about 36-fold higher compared to the drug solubility. The physicochemical characterization of this formulation confirmed the interaction between HA and HDA. The stability of this ionic interaction will require further studies, although preliminary evaluation suggested a limited influence of the pH in the range between 4.0 and 6.0 here considered. Therefore, the obtained polymeric micelles can be considered as a promising carrier for poorly soluble drugs, especially envisaging cutaneous or vaginal application.

## Figures and Tables

**Table 1:** Mixture components and constraints.

Component	Composition Limits	
	Minimum Amount (% w/w)	Maximum Amount (% w/w)
Hyaluronic Acid (HA)	56	80
Hexadecylamine (HDA)	4	33
Cholesterol (CHOL)	10	22

**Table 2:** Mean dimensions (particle size, PS) and polydispersion index (PI) of HA-based ionic micelles, unloaded and loaded with pyrene probe. DDA: dodecyl amine, HDA:hexadecylamine.

	Unloaded Micelles		Pyrene Loaded Micelles	
	PS (nm)	PI	PS (nm)	PI
HA/DDA	427 (± 47)	0.67 (± 0.23)	487 (± 35)	0.90 (±0.29)
HA/HDA	419 (± 8.6)	0.75 (± 0.015)	204 (± 17)	0.39 (± 0.05)

**Table 3:** Intensity of characteristic emissions of pyrene spectrofluorimetric spectrum in distilled water and encapsulated in HA/DDA and HA/HDA micelles. R_III/I_ represents the ratio of I_372_ and I_383_ emission values.

	I_372_	I_383_	R_III/I_
Water	219.85	146.99	0.67
Micelles HA/DDA	164.10	119.12	0.73
Micelles HA/HDA	346.15	317.41	0.87

**Table 4:** ANOVA table for the effects of the studied factors and their interactions on micelle dimensions.

Source	Sum of Squares	Df	Mean Square	F-Ratio	*p*-Value
A: HA/HDA ratio	528.12	1	528.12	4.22	0.10
B: CHOL%	6.48	1	6.48	0.05	0.831
C: Temperature	480.5	1	480.5	3.84	0.122
AB: HA/HDA ratio: CHOL%	4.21	1	4.21	0.03	0.863
AC: HA/HDA ratio:Temperature	146.21	1	146.21	1.17	0.340
**BC: CHOL%:Temperature**	**1635.92**	**1**	**1635.92**	**13.08**	**0.022**
Total error	500.11	4	125.03		
Total (corr.)	3301.54	10			

In bold the significant effects; R-squared = 84.852%; R-squared (adjusted for d.f.) = 62.131%.

**Table 5:** ANOVA table for the effects of the studied factors and their interactions on CLO concentration.

Source	Sum of Squares	Df	Mean Square	F-Ratio	*p*-Value
A: HA/HDA ratio	10.465	1	10.465	4.14	0.112
**B: CHOL%**	27.343	1	27.343	10.81	**0.030**
C: Temperature	7.508	1	7.5078	2.97	0.160
**AB: HA/HDA ratio: CHOL%**	38.852	1	38.852	15.36	**0.017**
**AC: HA/HDA ratio: Temperature**	22.412	1	22.412	8.86	**0.041**
**BC: CHOL%: Temperature**	22.412	1	22.412	8.86	**0.041**
Total error	10.118	4	2.529		
Total (corr.)	139.109	10			

In bold the significant effects; R-squared = 92.726%; R-squared (adjusted for d.f.) = 81.817%.

**Table 6:** Model validation experiments (Experiment). Comparison between predicted and experimental CLO concentration.

Exp#	HA	HDA	CHOL	CLO Predicted ± CI (95%) (µg/mL)	CLO Concentration Found (*) (µg/mL)	Relative Error (**) (%)
1	0.570	0.330	0.100	17.6 ± 4.9	18.37	4
2	0.560	0.270	0.170	10.2 ± 3.9	9.23	−10
3	0.660	0.210	0.130	6.1 ± 4.0	6.67	8
4	0.800	0.040	0.160	9.2 ± 4.3	10.06	8
5	0.590	0.190	0.220	4.7 ± 3.3	5.23	11

* Mean value of two replicate analyses; ** $\;Relative\;Error=100\times \frac{\left[CLO\right]found-\left[CLO\right]predicted}{\left[CLO\right]found}$.

## Supplementary Materials

The following are available online at https://www.mdpi.com/2079-4991/10/4/635/s1, Table S1: Full factorial and central composite design experimental matrix; Table S2: Mixture design experimental matrix.
