# Supplementary material for: Design of Experiments-Assisted Development of Clotrimazole-Loaded Ionic Polymeric Micelles Based on Hyaluronic Acid
Source: Nanomaterials (Basel). 2020 Mar 29;10(4):635. doi: 10.3390/nano10040635 (PMC7221810; doi:10.3390/nano10040635)
Supplement: Supplementary file 1 [file nanomaterials-10-00635-s001.pdf]

Supplementary Materials: Design of Experiments-Assisted Development of Clotrimazole-Loaded Ionic Polymeric Micelles Based on Hyaluronic Acid

Laura Catenacci †, Giorgio Marrubini †, Milena Sorrenti, Silvia Rossi, Giuseppina Sandri, Franca Ferrari, Valentina Fagnani, Caterina Valentino and Maria Cristina Bonferoni *

Department of Drug Sciences, University of Pavia, Viale Taramelli 12, Pavia 27100, Italy; laura.catenacci@unipv.it (L.C.), giorgio.marrubini@unipv.it (G.M.), milena.sorrenti@unipv.it (M.S.), silvia.rossi@unipv.it (S.R.), giuseppina.sandri@unipv.it (G.S.), franca.ferrari@unipv.it (F.F.), valentina.fagnani01@universitadipavia.it (V.F.), caterina.valentino@universitadipavia.it (C.V.)

* Correspondence: cbonferoni@unipv.it; Tel.: +39-0382-987375

† These two authors equally contributed to the paper.

Central Composite Design of experiments was computed adding 8 points to the Full Factorial Design of experiments used in the screening phase of the study (Experiments # 1–11). The selected additional points were 6 star points at coded coordinates of (±1.68, 0, 0), (0, ±1.68, 0), and (0, 0, ±1.68) corresponding to experiments # 12–17 in the Table S1.

Table S1. Full factorial and central composite design experimental matrix.

	X_1 HA/HDA	X_2 CHOL %	X_3 Temperature	HA/HDA (molar ratio)	CHOL (%, w/w HA)	Temperature (°C)	Response Clotrimazole (µg/mL)
1	−1	−1	−1	1:0.75	30	40	1.13
2	1	−1	−1	1:0.25	30	40	1.83
3	−1	1	−1	1:0.75	10	40	3.24
4	1	1	−1	1:0.25	10	40	13.81
5	−1	−1	1	1:0.75	30	20	5.36
6	1	−1	1	1:0.25	30	20	0.42
7	−1	1	1	1:0.75	10	20	1.83
8	1	1	1	1:0.25	10	20	4.65
9	0	0	0	1:0.50	20	30	6.06
10	0	0	0	1:0.50	20	30	7.65
11	0	0	0	1:0.50	20	30	4.83
12	−1.68	0	0	1:0.08	20	30	10.06
13	+1.68	0	0	1:0.92	20	30	9.90
14	0	−1.68	0	1:0.50	3.18	30	0.67
15	0	+1.68	0	1:0.50	36.82	30	1.24
16	0	0	−1.68	1:0.50	20	13	0.85
17	0	0	+1.68	1:0.50	20	47	2.16

Table S2. Mixture design experimental matrix.

Exp#	X_1: HA (w/w)	X_2: HDA (w/w)	X_3: CHOL (w/w)	CLO Concentration (μg/mL)
1	0.570	0.330	0.100	18.12
2	0.800	0.100	0.100	12.45
3	0.560	0.220	0.220	6.70
4	0.740	0.040	0.220	4.94
5	0.685	0.215	0.100	6.64
6	0.560	0.275	0.165	10.07
7	0.800	0.040	0.160	8.61
8	0.660	0.210	0.130	7.65
9	0.590	0.190	0.220	5.23
